# Comparative survival of five porcine reproductive and respiratory syndrome virus strains on six fomites

**DOI:** 10.14202/vetworld.2024.2774-2779

**Published:** 2024-12-13

**Authors:** Angie Quinonez-Munoz, Nader M. Sobhy, Sagar M. Goyal

**Affiliations:** 1 Department of Veterinary Population Medicine, University of Minnesota, St. Paul, Minnesota, USA; 2 Department of Animal Medicine, Faculty of Veterinary Medicine, Zagazig University, Zagazig, Sharkia, Egypt

**Keywords:** fomites, porcine reproductive and respiratory syndrome virus, survival, swine, viability, viral strains

## Abstract

**Background and Aim:**

Despite the availability of vaccines, porcine reproductive and respiratory syndrome virus (PRRSV) continues to cause disease outbreaks in pigs worldwide. One of the reasons for this problem is the frequent mutation of the virus, which creates new variants. This study was conducted to determine the survival of five PRRSV strains on four non-porous and two porous fomites at 22–25°C (room temperature).

**Materials and Methods:**

Five strains of PRRSV (1-7-4, 1-8-4, VR 2332, 1-4-4 MN, and 1-4-4 SD) were used in this study. Circular pieces of aluminum, boot material, polyvinyl chloride, stainless steel, cardboard, and concrete were used as fomites. A small volume of each virus strain was placed on the fomite, followed by incubation at room temperature. The virus surviving at different time points was eluted in an eluent solution. Serial 10-fold dilutions of the eluate were inoculated in MARC-145 cells for virus titration. Multivariate analysis of variance (MANOVA) was used for statistical analysis, and *post hoc* analysis was used for multiple pairwise comparisons.

**Results:**

Three of the five strains were inactivated within 36 h on non-porous fomites; the remaining two survived for 72 h. On porous fomites, all five strains were inactivated within 12 h. MANOVA at p < 0.05 indicated that the inactivation of strains 1-7-4 and 1-4-4 SD was significant compared with the other strains. In addition, the number of virus titers was significantly reduced on stainless steel compared to other fomites.

**Conclusion:**

Our findings illustrate how the interaction between the PRRSV strain and fomite material affect viral stability over time. The results also provide an understanding of fomites’ role in PRRSV epidemiology as indirect transmitters of the virus.

## Introduction

Frank outbreaks and endemic infections caused by porcine reproductive and respiratory syndrome virus (PRRSV) continue to cause heavy economic losses to the swine industry worldwide [1, 2]. Financial losses due to this disease in the U.S. have been estimated at $664 million annually [3]. The virus is a small, enveloped, and single-stranded RNA virus belonging to the family *Arteriviridae* and order *Nidovirales* [4]. PRRSV-1 (type strain Lelystad) and PRRSV-2 (type strain VR-2332) are two species currently recognized by the International Committee on Taxonomy of Viruses [5]. Several subtypes, isolates, and variants have appeared within each PRRSV species over the past 30 years due to high mutation rates, selection, and recombination. The wide diversity of viral strains has led to many challenges in the diagnosis, epidemiology, and control of this virus [6]. Animals infected with PRRSV shed the virus in their saliva, nasal secretions, urine, semen, mammary secretions, and feces, leading to direct virus transmission within and between pig populations [7, 8]. In addition, these excretions and secretions can contaminate facilities, personnel, and equipment on the farm and become a potential source for the indirect transmission of viruses through fomites.

Fomites (or inanimate objects) can be non-porous or porous. Porous fomites include boots, fabric, cardboard, and coveralls [9]. Factors influencing virus survival in the environment include the fomite material, moisture, pH, and temperature. The PRRSV was stable at pH 6.5–7.5. Jacobs *et al*. [10] have addressed the stability of PRRSV at ~22–25°C (room temperature). Jacobs *et al*. [10] observed significant differences in the half-life of four PRRSV-2 isolates (VR 2332, JA-142, MN 184, and Ingelvac vaccine virus) at different temperatures; a mean half-life of 27.4 h (range 16.9 h–37.9 h; 95% confidence interval) was reported at 20°C. Pirtle and Beran [11] studied the survival of PRRSV on fomites at room temperature; the authors were not able to detect infectious PRRSV on dry materials (e.g., plastic, stainless steel, rubber, alfalfa, wood shavings, straw, corn, swine starter feed, or denim cloth) beyond the day of inoculation at 25°C–27°C. In a study by Dee *et al*. [12], the MN 30–100 strain of PRRSV was detectable by RT-PCR after 8 h on plastic, metal, cardboard, Styrofoam, linoleum, and rubber at 20°C. However, different results were obtained when virus isolation was done in cell cultures; viable virus was detected for up to 8 h on plastic, 4 h on rubber, and 2 h on metal and Styrofoam. No viable virus was recovered from cardboard and linoleum. These data are concerning for the swine industry because outbreaks continue to occur, and new variants emerge on an almost annual basis.

A more recent study by Munoz *et al*. [13] evaluated the comparative survival of 10 PRRSV strains at three different temperatures. We found that strains 1-8-4, 1-4-4, and VR 2332 survived on polystyrene at room temperature between 3 and 7 days. The half-life (T1/2) of strain 1-8-4 was higher than that of other strains, indicating higher stability at different temperatures.

No information is available on the survival of newly emergent strains of PRRSV on fomites at room temperature. Hence, this study aimed to determine the comparative survival of five PRRSV strains against six fomites at room temperature.

## Materials and Methods

### Ethical approval

The study was approved by the Institutional Biosafety Committee (2210-40476H).

### Study period and location

This study was conducted from August 2022 to April 2023 at the University of Minnesota Veterinary Diagnostic Laboratory (UMVDL), Saint Paul, Minnesota, USA.

### Viruses

In total, five PRRSV-2 strains were used in this study. The strains used were 1-7-4, 1-8-4, VR 2332, 1-4-4 MN, and 1-4-4 SD. The strain (1-4-4 SD) was kindly provided by Prof. Eric Nelson of South Dakota State University, Brookings, SD. All strains were propagated and titrated in MARC-145 cells using Eagle’s minimum essential medium (MEM, Corning, NY, USA) supplemented with 4% fetal bovine serum (Fisher Scientific, USA), 50 μg/mL neomycin (Sigma- Aldrich, USA), 1 μg/mL fungizone (Sigma-Aldrich), 150 IU/mL penicillin, and 150 μg/mL streptomycin (Sigma-Aldrich). Monolayers of these cells were prepared in 96-well microtiter plates for virus titration.

### Procedure

Circular pieces (approximately 1 cm^2^) of six different fomites were cut to fit into individual wells of Costar 24-well cell culture plates (Corning). Aluminum, boot material, polyvinyl chloride (PVC), and stainless steel are non-porous fomites, while cardboard and concrete are porous fomites. For each strain to be tested, three pieces of each fomite were placed in three wells, each of a 24-well plate, followed by applying 40 μL of the virus to each fomite. The plates were incubated at room temperature, as measured using an indoor thermometer, varied between 23°C and 25°C. The surviving virus was eluted from pieces of fomites after 12, 24, 36, 48, 60, and 72 h for non-porous materials and after 4, 8, 12, and 24 h for porous materials using 200 μL of elution buffer (3% beef extract-0.05 M glycine). Briefly, the eluent (elution buffer) was placed on the fomites, and the buffer was pipetted back and forth a few times to recover the surviving virus. The reason for using different time points for porous materials (as opposed to those for non-porous materials) was based on preliminary tests in which no virus was recovered from cardboard and concrete after 24 h (data not shown).

### Virus titration

The serial 10-fold dilutions of all eluates were prepared in a maintenance medium. All dilutions were inoculated into monolayers of MARC-145 cells prepared in 96-well plates using 3 wells/dilution. After inoculation, plates were incubated at 37°C under 5% CO_2_ and were examined daily under an inverted microscope for the appearance of virus-induced cytopathic effects. Fifty percent of the endpoints were recorded after 7 days of incubation. Virus titers were calculated using the Karber method [14] and expressed as log_10_ TCID_50_/100 μL. The percent virus inactivation was calculated using the formula (A-B/A) × 100, where A is the initial virus titer and B is the remaining virus titer.

### Statistical analysis

Multivariate analysis of variance (MANOVA) was used to determine significant differences (p < 0.05) in virus titer reduction in non-porous fomites. Half-life (T1/2) was calculated using the calculator (https://www.calculator.net/half-life-calculator.html). The *post hoc* Tukey analysis was applied for multiple pairwise comparisons.

## Results

The initial titers of all five viral strains on non-porous and porous fomites are shown in column 1 of Tables-1 and 2, respectively. The starting titers ranged between 10^3.83^ and 10^6.17^ TCID_50_/100 μL. Table-1 lists the titers of surviving viruses on non-porous fomites, while Table-2 lists these values for porous fomites. The half-lives of all strains on different fomites are shown in Table-3. Virus survival was generally lower on porous fomites than on non-porous fomites. Three of the five strains were inactivated on non-porous fomites within 24 h and 36 h. However, strains 1-7-4 were not completely inactivated after 48 h and 60 h on aluminum and PVC, respectively. Strain 1-8-4 was inactivated between 24 and 36 h on all non-porous fomites, except boot material, which remained viable for >72 h. Strain VR 2332 was inactivated after 24 h on all non-porous materials. Strains 1-4-4 MN and 1-4-4 SD were not detectable after 36 h on any of these materials.

**Table-1:** Titers of PRRSV strains on non-porous fomites at 22°C–25°C (room temperature).

Virus strain (initial titer)^A^	Fomite	Average viral titer (per cent virus inactivation) at indicated times ^B^					

		12 h	24 h	36 h	48 h	60 h	72 h
1-7-4 (6.17)	AL^C^	4.00 (99.32)	2.34 (99.98)	0.83 (99.999)	0.42 (99.999)	<1 (>99.99)	<1 (>99.99)
	BT	3.84 (99.53)	3.34 (99.85)	<1 (>99.99)	<1 (>99.99)	<1 (>99.99)	<1 (>99.99)
	PVC	3.67 (99.68)	2.83 (99.95)	1.17 (99.999)	<1 (>99.99)	1.00 (99.99)	<1 (>99.99)
	SS	3.50 (99.79)	2.34 (99.98)	0.42 (99.999)	<1 (>99.99)	<1 (>99.99)	<1 (>99.99)
1-8-4 (5.50)	AL	1.67 (99.985)	0.42 (99.999)	<1 (>99.99)	<1 (>99.99)	<1 (>99.99)	<1 (>99.99)
	BT	2.00 (99.968)	1.50 (99.99)	0.75 (99.9997)	1.50 (99.99)	0.75 (99.9997)	1.50 (99.99)
	PVC	2.00 (99.968)	0.42 (99.999)	<1 (>99.99)	<1 (>99.99)	<1 (>99.99)	<1 (>99.99)
	SS	1.67 (99.985)	0.42 (99.999)	<1 (>99.99)	<1 (>99.99)	<1 (>99.99)	<1 (>99.99)
VR 2332 (4.50)	AL	2.50 (99.00)	0.00 (>99.99)	<1 (>99.99)	<1 (>99.99)	<1 (>99.99)	<1 (>99.99)
	BT	2.50 (99.00)	0.59 (99.987)	<1 (>99.99)	<1 (>99.99)	<1 (>99.99)	<1 (>99.99)
	PVC	3.00 (96.83)	1.34 (99.93)	<1 (>99.99)	<1 (>99.99)	<1 (>99.99)	<1 (>99.99)
	SS	2.83 (97.86)	0.42 (99.991)	<1 (>99.99)	<1 (>99.99)	<1 (>99.99)	<1 (>99.99)
1-4-4 MN (6.17)	AL	2.83 (99.95)	<1 (>99.99)	<1 (>99.99)	<1 (>99.99)	<1 (>99.99)	<1 (>99.99)
	BT	3.17 (99.90)	0.92 (9.999)	<1 (>99.99)	<1 (>99.99)	<1 (>99.99)	<1 (>99.99)
	PVC	3.17 (99.90)	1.09 (99.999)	<1 (>99.99)	<1 (>99.99)	<1 (>99.99)	<1 (>99.99)
	SS	3.00 (99.93)	0.42 (99.999)	<1 (>99.99)	<1 (>99.99)	<1 (>99.99)	<1 (>99.99)
1-4-4 SD (3.83)	AL	0.75 (99.92)	<1 (>99.99)	<1 (>99.99)	<1 (>99.99)	<1 (>99.99)	<1 (>99.99)
	BT	1.17 (99.78)	0.92 (99.88)	<1 (>99.99)	<1 (>99.99)	<1 (>99.99)	<1 (>99.99)
	PVC	1.50 (99.53)	1.09 (99.82)	<1 (>99.99)	<1 (>99.99)	<1 (>99.99)	<1 (>99.99)
	SS	0.75 (99.92)	0.42 (99.96)	<1 (>99.99)	<1 (>99.99)	<1 (>99.99)	<1 (>99.99)

A Titers are expressed as log_10_ TCID_50_/100 μL.

B Limit of detection: 1 log_10_ TCID_50_/100 μL.

C AL=Aluminum, BT=Boot material, SS=Stainless steel, PVC=polyvinyl chloride, PRRSV=Porcine reproductive and respiratory syndrome virus

**Table-2:** Titers of PRRSV strains on porous fomites at 22°C–25°C (room temperature).

Virus strain (initial titer)^A^	Fomite	Average viral titer (per cent virus inactivation) at indicated times^B^			

		4 h	8 h	12 h	24 h
1-7-4 (6.17)	CB^C^	2.83 (99.95)	1.17	0.42	<1 (>99.99)
	CT	0.42 (99.999)	(99.999) <1 (>99.99)	(99.999) <1 (>99.99)	<1 (>99.99)
1-8-4 (5.50)	CB	(99.996)	<1 (>99.99)	<1 (>99.99)	<1 (>99.99)
	CT	<1 (>99.99)	<1 (>99.99)	<1 (>99.99)	<1 (>99.99)
VR 2332 (4.50)	CB	2.33 (99.32)	0.42 (99.99)	<1 (>99.99)	<1 (>99.99)
	CT	<1 (>99.99)	<1 (>99.99)	<1 (>99.99)	<1 (>99.99)
1-4-4 MN (6.17)	CB	2.83 (99.95)	1.50	(99.999)	<1 (>99.99)
	CT	0.42 (99.999)	(99.997) <1 (>99.99)	<1 (>99.99)	<1 (>99.99)
1-4-4 SD (3.83)	CB	<1 (>99.99)	<1 (>99.99)	<1 (>99.99)	<1 (>99.99)
	CT	<1 (>99.99)	<1 (>99.99)	<1 (>99.99)	<1 (>99.99)

A Titers are expressed as log_10_ TCID_50_/100 μL.

B Limit of detection: 1 log_10_ TCID_50_/100 μL.

C CB=Cardboard, CT=Concrete, PRRSV=Porcine reproductive and respiratory syndrome virus

**Table-3:** Half-life (t1/2) and mean half-life (τ) of PRRSV strains on different fomites.

Virus strain	half-life, t1/2 in hours (mean lifetime, τ) on indicated fomite					

	Aluminum	Boot material	Cardboard	Concrete	PVC	Stainless steel
1-7-4	12.38 (17.86)	27.10 (39.10)	3.09 (4.46)	1.03 (1.48)	15.00 (21.65)	9.28 (13.39)
1-8-4	6.46 (9.33)	38.41 (55.41)	1.62 (2.34)	NC	6.46 (9.33)	6.46 (9.33)
VR2332	14.15 (20.41)	8.18 (11.81)	2.33 (3.37)	NC	13.73 (19.81)	7.01 (10.11)
1-4-4 MN	10.67 (15.39)	8.74 (12.61)	4.57 (6.59)	1.03 (1.48)	9.59 (13.84)	11.53 (16.64)
1-4-4 SD	5.10 (7.35)	7.01 (10.11)	NC	NC	8.87 (12.80)	5.10 (7.35)

NC=Not calculated, PVC=polyvinyl chloride, PRRSV=Porcine reproductive and respiratory syndrome virus

None of the five strains were detectable on porous cardboard after 24 h. However, there were some differences in their survival. For example, 1-7-4 and 1-4-4 MN survived the longest (up to 12 h) on cardboard. Strain 1-4-4 SD, on the other hand, was the least resistant; it was not recovered from cardboard and concrete after 4 h. None of the strains survived on concrete for more than 4 h.

Table-3 presents the half-lives of all strains. The highest survival on aluminum, boot material, PVC, stainless steel, cardboard, and concrete was for VR 2332, 1-8-4, 1-7-4, 1-4-4 MN, 1-4-4 MN, and 1-4-4 MN, respectively. The use of MANOVA at p < 0.05 indicated significance about time (F = 5.24) and fomite (F = 3.24). There was an interaction between the virus and time at F (18.24) and between the virus and fomite at F (10.24). The inactivation of 1-7-4 and 1-4-4 SD was significant compared with the other strains. The titer was significantly reduced in the stainless steel compared with the other fomites. The virus titers were significantly decreased after 12 h and 24 h compared with the other time points.

## Discussion

Enveloped viruses such as influenza, pox, and rabies cause numerous disease outbreaks in humans and animals. The recent outbreaks and misery caused by SARS-CoV-2 necessitate that data on the stability of viruses in the environment be available to develop appropriate control methods [15]. Enveloped viruses (including PRRSV) may settle on different surfaces and remain viable and infectious for a period of time, thereby posing a risk of exposure to in-contact animals [15, 16]. Factors influencing the survival of viruses on surfaces include strain type, initial virus titer, surface type, virus-suspending medium, mode of deposition, temperature, relative humidity, and method used to determine the viability of the virus [17].

Casanova *et al*. [18] studied the effects of temperature on the survival of two coronaviruses, for example, transmissible gastroenteritis virus (TGEV) and mouse hepatitis virus (MHV). They found that the infectious virus persisted for 28 days at 4°C and that low relative humidity (~20%) favored virus survival for longer. In a classic study, the survival of PRRSV in various vehicles/fomites was investigated at 25°C–27°C [11]; the authors found that PRRSV survived for up to 11 days in drinking water but was inactivated within 1 day when applied to alfalfa, wood shavings, straw, plastic, boot rubber, and stainless steel.

This study investigated the survival at room temperature (22°C–25°C) of five different strains of PRRSV-2 on non-porous and porous fomites. The most common materials found on pig farms were selected as test fomites. For example, aluminum and PVC are commonly used as barriers and doors in swine farms. Our results indicate that the virus survives longer on aluminum than PVC, which is used routinely in farrowing houses and nurseries because it is comfortable, non-corrosive, and easy to clean. If cleaned and disinfected regularly, the exposure of animals to pathogens and piglet mortality can be reduced [19].

Stainless steel is preferred in feeders and drinkers due to its rust-resistance properties. The viruses on this material did not survive for a long time. Similarly, several strains did not survive for a long time on boot material. These results are similar to those reported by Otake *et al*. [9], who could not detect any virus on boots after 2 days. It has been demonstrated that PRRSV can contaminate boots and coveralls of animal handlers and that these contaminated fomites can transmit the infection to naïve pigs. However, the use of sanitation protocols appeared to limit virus transmission [9].

Cardboard boxes containing medicines, vaccines, and supplements are frequently used in swine farms. Sometimes, empty boxes can get trampled along with waste and manure and be turned into fertilizer. This practice seems dangerous because viruses can survive on cardboard for a long time due to their porous nature. Elution of the virus from cardboard for up to 12 h indicates the importance of this fomite in virus transmission.

Concrete is the standard farm structure for raising pigs. It has many advantages, as it is easier to manage, wash, and disinfect. However, concrete is not amenable to pig behavior since they cannot root, dig, and shower with mud to control their body temperature. The reduction of the virus in concrete may occur due to fluid’s rapid absorption from concrete, exposing the virus to more dryness. On the other hand, the virus may enter the fine pores of concrete and is difficult to elute after loading, which may lead to false-positive results.

The composition of fomite and its porosity may influence virus survival [20]; they studied the survival of the porcine epidemic diarrhea virus on Styrofoam, rubber, plastic, coveralls, aluminum foil, and cardboard. At 4°C, the virus survived for 5–15 days. The authors concluded that the type of fomite material and temperature may affect the stability of this virus.

In agreement with Pirtle and Beran [11], our results showed lower stability of PRRSV strains on porous materials. Corpet [21] classified fomites based on the length of survival of SARS-CoV-2. In order of long to short stability, the list contained polypropylene (mask), plastic, glass, stainless steel, pig skin, cardboard, banknote, cotton, wood, paper, tissue, and copper. The authors observed that moisture absorption by porous materials may inactivate the virus more readily and that smooth and waterproof materials may protect against inactivation [21]. Dryness has been observed to rapidly inactivate the virus as it leads to alterations in the bilayer membrane that may need water for balance [17, 18].

Jacobs *et al*. [10] studied the survival of four different strains of PRRSV suspended in cell culture medium at 4°C, 10°C, 20°C, and 30°C. They concluded that temperature, but not virus strain, significantly affected the half-life of the virus. In contrast, our results indicate differences in the survival of viral strains, although at a very low level. For example, our findings show that PRRSV strain 1-7-4 exhibits higher survivability than other strains. Since 2016, this strain has been associated with several clinical cases of dramatic abortion storms with high mortality in piglets. It is known that the pathogenicity of isolates belonging to the same RFLP type can vary [22]. For example, strain 1-8-4 was recovered from an outbreak in Minnesota in 2001 and classified as a highly pathogenic PRRSV isolate [23]. This strain showed the highest survival (up to 72 h) on boot material in this study. Thus, disinfection and/or changing boots before entering the farm (and for moving between barns) are essential.

The strain VR2332 was first characterized and isolated in 1992. The virus infectivity of CL2621 cells was reduced by 50% after incubation for 12 h at 37°C and was completely inactivated after 48 h of incubation at 37°C and 45 min at 56°C [24]. Our results on non-porous fomites show that the half-life of this strain on aluminum and PVC was the highest compared with other strains (for up to 36 h). In a viability study of strain MN-30100, viable virus was recovered from plastic after 8 h, metal and Styrofoam after 2 h, and rubber after 4 h. No virus was recovered from the cardboard and linoleum after 1 h [12]. Although this study was performed at a lower temperature than the present study, a different strain was evaluated, and no infectivity data were presented; however, it provides some insights into the survivability of PRRSV on porous materials. The absence of virus detection on cardboard after a short time is consistent with our findings.

The regional outbreak caused by PRRSV strain 1-4-4 L1C was reported in 2021. This strain caused high production losses, mainly in growing pigs [25]. The virus was inactivated at 36 h in boots material and PVC and at 24 h in aluminum and stainless steel. Half-lives were similar on boots material and PVC and on aluminum and stainless steel. Strain 1-4-4 SD was inactivated after 24 h in all non-porous fomites and after 4 h on porous fomites. The differences in the stability of these related strains may be attributed to genetic variations. The relationship between genotypic and phenotypic stability is still unclear and requires further research.

One limitation of this study is that it was conducted on clean fomites. If fomites were contaminated with organic matter, the virus survival may have been prolonged. Another limitation is the low number of replicates, which prevents us from drawing conclusions from the statistical analysis. However, this study provides insights into the survival of PRRSV on different types of fomites at room temperature. The persistence of viruses on fomites for long periods and the housing temperature of swine farms constitute important risk factors for the transmission of PRRSV. In addition, temperature fluctuations in swine farms located in the northern part of the United States contribute to the complexity of prevention and control programs for PRRSV. Therefore, biosecurity practices, such as proper disinfection and drying of supplies or equipment consisting of the materials evaluated in this study, are critical and should be adhered to since the virus can survive at room temperature for hours to a few days. Despite the shorter survival of PRRSV on porous materials (cardboard and concrete), these fomites should also be considered when designing disinfection protocols.

## Conclusion

Our findings provide a perspective on how the interaction between the PRRS virus strain and fomite material affects viral stability over time. This study provides an understanding of fomites’ role in PRRSV epidemiology as indirect transmitters of the virus. Although previous studies have illustrated the survivability of PRRSV on fomites, our study compared the survivability of different strains of the virus on different fomites surfaces. Data analysis revealed significant variations in survivability among strains from different fomites. Titer reduction in the 1-7-4 and 1-4-4 SDs was significant compared with the other strains. The titer was significantly reduced in stainless steel compared with the other fomites. These results should be useful in the design of control and biosecurity measures.

## Authors’ Contributions

SMG and NMS: Conceptualization of the study and drafted and revised the manuscript. AQ and NMS: Methodology and curation and drafted the manuscript. SMG: Supervision and project administration. All authors have read and approved the final manuscript.
